# Need for Echocardiographic Analysis of Abdominal Aorta Dimensions and Their Associations with Demographic Characteristics in Healthy Individuals

**DOI:** 10.31661/gmj.v13i.3259

**Published:** 2024-06-28

**Authors:** Haleh Bodagh, Kamran Mohammadi, Asma Yousefzadeh, Mehran Rahimi, Mehrnoush Toufan-Tabrizi

**Affiliations:** ^1^ Cardiovascular Research Center, Tabriz University of Medical Sciences, Tabriz, Iran

**Keywords:** Abdominal Aorta, Aneurysm, Aortic Aneurysm, Abdominal, Healthy Individuals

## Dear Editor,

Abdominal aortic aneurysms (AAA) pertain to dilations in the abdominal aorta. A practical definition of an abdominal aortic aneurysm is a transverse diameter of 3 cm or more [1]. In the general population, the prevalence of AAA is observed in males at a prevalence ranging from 5.3% to 6.7%, while in females, the prevalence falls within the range of 1.2% to 1.9% [2]. Most affected individuals are unaware of their condition [3]. Aortic aneurysms rank as the 14th leading cause of death in the United States, with numerous fatalities occurring annually due to ruptured AAAs or preventive procedures [4]. Various risk factors significantly elevate the likelihood of developing an abdominal aortic aneurysm, including age over 60, smoking, and high blood pressure [5]. The probability of aneurysm rupture is influenced by factors such as aneurysm size, rate of expansion, smoking, and persistent hypertension [6].

Most abdominal aortic aneurysms are asymptomatic and are incidentally detected through imaging techniques such as ultrasound, computed tomography, or magnetic resonance imaging, presenting challenges for screening. Despite these challenges, the US Preventive Services Task Force recommends abdominal ultrasonography screening for men between the ages of 65 and 75 with a history of smoking [7]. Moreover, age, gender, height, weight, body mass index (BMI), and body surface area (BSA) are among multiple factors that have been shown to significantly associate with the size of the abdominal aorta [8]. Although studies have explored the diameter of the abdominal aorta in some populations, determining the normal diameter of the abdominal aorta in diverse populations, its correlation with demographic factors, and ultimately a normal range in healthy people is crucial for diagnosing and managing abdominal aortic diseases [9].

The routine assessment of the proximal abdominal aorta alongside the inferior vena cava (IVC) is a common practice for echocardiologists [10]. Despite being often used in the clinical setting, this aspect of echocardiography may not have received adequate attention. Many individuals undergo routine echocardiographic assessment of the proximal abdominal aorta at medical facilities, presenting a valuable opportunity for early diagnosis. To achieve this, precise measurements of the abdominal aorta’s size in normal individuals and its relationship with other aortic segments need accurate determination. There is a need for studies that aim to contribute to the broader understanding of abdominal aortic diameters by shedding light on the size of the abdominal aorta in a healthy population.

In conclusion, we emphasize the critical need for an echocardiographic analysis of abdominal aorta dimensions and their associations with demographic characteristics in healthy individuals. Also, we suggest using age, gender, and BSA to be included and analyzed in healthy individuals in the future studies as they are the most important demographic and anthropometric factors in AAA [11].

## Conflict of Interest

None.
